# Pulmonary mucosa-associated lymphoid tissue lymphoma with Sjögren’s syndrome and literature review: A case report

**DOI:** 10.1097/MD.0000000000035232

**Published:** 2023-09-15

**Authors:** Limin Yang, Rongfeng Qu, Fang Liu, Chunmei Hu

**Affiliations:** a Department of Hematology and Oncology, Second Hospital of Jilin University

**Keywords:** associated lymphoid tissue lymphoma, case report, Hodgkin’s lymphoma, non, Pulmonary mucosa, Sjögren’s syndrome

## Abstract

**Introduction:**

A 54-year-old woman was admitted to hospital with chest tightness, shortness of breath, and chest pain on exertion. Her chest computed tomography showed a space-occupying lesion in the right lower lobe of the lung.

**Case presentation:**

The ultrasound-guided right lung mass biopsy showed mucosa-associated lymphoid tissue (MALT), and the patient was diagnosed with Sjögren’s syndrome (SS). The patient’s symptoms were partially relieved with chemotherapy.

**Conclusions:**

Autoimmune diseases like SS and systemic lupus erythematosus are recognized risk factors for pulmonary MALT. It is necessary to identify predictors of malignant transformation of SS to pulmonary MALT lymphoma.

## 1. Introduction

Mucosa-associated lymphoid tissue (MALT) is a lymphoid tissue specializing in the defense of the mucosa. It was first described in the gastrointestinal tract in animal models and subsequently in the ileum in humans. MALT lymphoma is a distinct subtype of non-Hodgkin’s lymphoma (NHL) in which any mucosal site can be affected. The most common primary site of MALT lymphoma is the stomach (45%), followed by the conjunctiva (14%) and orbit (11%). Pulmonary MALT lymphoma is relatively uncommon and was first reported by Herbert in 1983.^[1]^ Disorders involving chronic antigen stimulation, including systemic lupus erythematosus, multiple sclerosis, Hashimoto thyroiditis, and Sjögren’s syndrome (SS) are all recognized risk factors for developing pulmonary MALT lymphoma.^[2]^ Computed tomography (CT) of the chest, abdomen, and pelvis and/or whole-body positron-emission tomography CT scans are also usually obtained. Apart from gastric MALT lymphoma, staging is performed using the Ann Arbor Staging system.^[3,4]^ The relative rarity and heterogeneity of patients with pulmonary MALT lymphoma, as well as their different biology, clinical presentation, and behavior, make it difficult to determine the best treatment for these patients. Currently available treatments include surgical resection, chemotherapy, immunotherapy, radiation therapy, and observation. However, the optimal treatment regimen remains to be determined. We herein present the case of a 55-year-old patient diagnosed with pulmonary MALT lymphoma and SS, as well as a literature review on the current state of research and treatment of these diseases.

## 2. Case report

**Figure 1.**
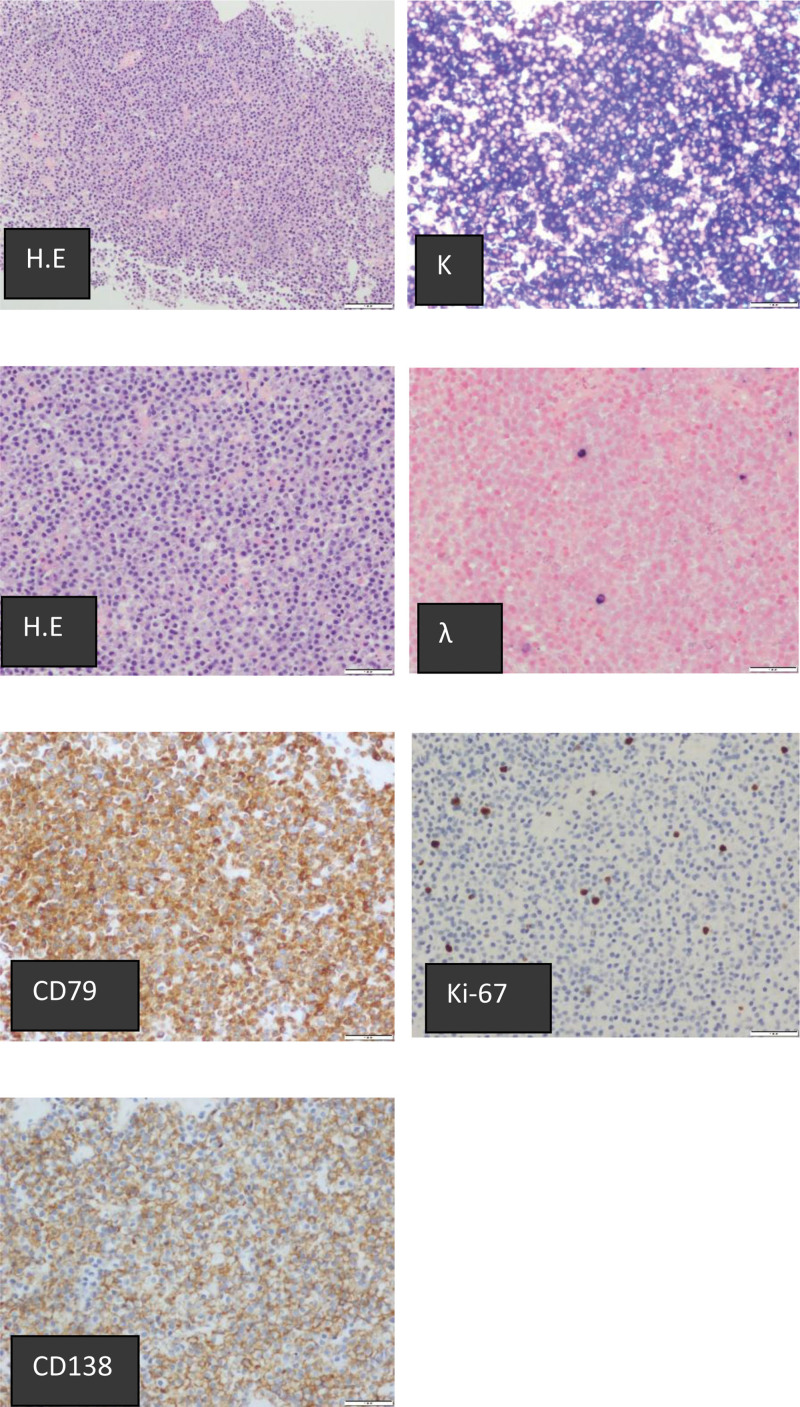
Histological findings for right lung mass with hematoxy-in and eosin staining (original magnification, ×4, ×400) and immunostaining at diagnosis of MALT. Immunohistochemically, abnormal lymphocytes are positive for CD79, CD138, and κ and negative for CD20.

A 54-year-old female patient developed chest tightness and shortness of breath following activity 2 years ago and occasionally coughed, with yellow, sticky sputum that was difficult to expel. The patient did not demonstrate any fever or receive systemic treatment prior to hospitalization. Despite subsequent recurrence of the same symptoms, the patient did not receive any systematic treatment. Half a month prior to admission, the symptoms recurred, with no obvious trigger. Right chest pain occurred upon performing light activity but could be gradually relieved with rest. Chest CT in the local hospital showed the lower lobe of the right lung was occupied, and the upper lobe of the left lung had nodular high-density opacity. After considering the upper and middle lobes and left pneumonia of the right lung, the patient received symptomatic anti-infective drugs. She reported that her symptoms did not significantly improve; thus, she was treated at the Second Hospital of Jilin University for further diagnosis and treatment. Physical examination showed coarse breath sounds in both lungs, weak breath sounds in the right lower lung, and a small number of crackles at the base of the right lung. Laboratory tests demonstrated the following results: white blood cell count, 2.5 × 10^9^/L; neutrophil count, 1.61 × 10^9^/L; hemoglobin level, 91 g/L; and β2-microglobulin count, 6.15 mg/L. Blood gas analysis without oxygen revealed the following results: pH, 7.45; PCO_2_, 37 mm Hg; PO_2_, 53 mm Hg; SaO_2_, 89%; immunoglobulin G levels, 19.8 g/L; immunoglobulin A levels, 52.5 g/L; complement C3 levels, 53.5 mg/dL; complement C4 levels, 14.3 mg/dL; SS A antibody (WB) status, positive (+++); 52 kDa protein antibody (WB) antibody status, positive (+++); and ribosomal P protein antibody (WB) status, weakly positive (+–). Antinuclear antibody (ANA) screening (IIF) revealed a ratio of 1:320 and an ANA fluorescence model nuclear particle type. Lip gland (lower lip) biopsy revealed multifocal lymphocytes around the mucus gland of the lip gland, with each foci being >50 lymphocytes. Ultrasound-guided right lung mass aspiration biopsy was performed, and the pathology revealed diffuse proliferation of plasmoid cells. The cells had a plasma cell phenotype and light chain restricted expression, which combined with immunohistochemical staining results to support non-Hodgkin’s B-cell lymphoma and plasma cell differentiation, leading us to suspect MALT lymphoma. Immunohistochemistry results were as follows: CD10 part (+), CD79a (+), Bcl-2 (+), CD3, CD5, CD20, CD56, Bcl-6, and cyclin D1 (–), Kappa (light chain restrictive expression), and Lambdn (light chain restrictive expression) (Fig. 1).

**Figure 2.**
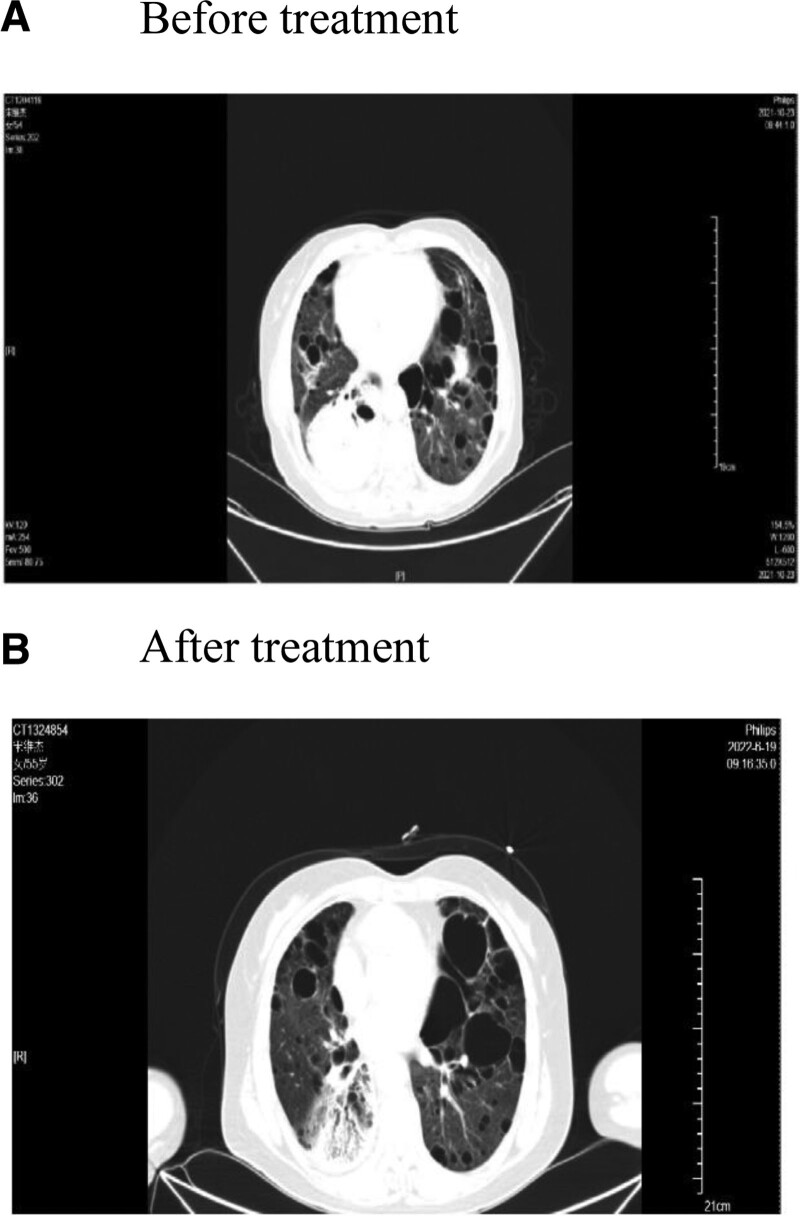
Computed tomography scan shows that the soft tissue density mass in the lower lobe of the right lung was flaky and had a slight high-density shadow. (A) Before treatment and (B) after treatment.

Subsequent positron-emission tomography CT showed that the soft tissue density mass in the lower lobe of the right lung was flaky and had a slight high-density shadow of approximately 90 × 75 × 120 mm in size. The maximum standardized uptake value was 13.2, and the multiple flaky and slight high-density opacities in both lungs were consistent with lymphoma accompanied by intrapulmonary invasion. Accordingly, the tumor stage was considered to be stage IVB according to the Ann Arbor classification of lymphoma. After a clear diagnosis was reached, the patient received 3 cycles of CHOP (cyclophosphamide, doxorubicin, vincristine, prednisolone) treatment starting October 2021. After combining the patient’s blood M protein, IGM-Kappa type persisted, globulin levels were >40 g/L, and a second pathology biopsy still showed obvious plasma cell differentiation. Accordingly, the R-CHOP regimen was administered for 4 cycles. Repeat examination after 6 cycles of chemotherapy showed that the SPD of intrapulmonary lesions was reduced by ≥50% (Fig. 2). The patient was considered to have undergone partial remission based on the evaluation criteria of the treatment effect on Lugano lymphoma.

### 2.1. Consent and ethics

Ethical approval for this study was provided by the Ethics Committee of the Second Hospital of Jilin University, China, on May 18, 2023.

## 3. Discussion

M proteins and a free monoclonal immunoglobulin IgM κ light chain were observed on serum protein electrophoresis and blood immunofixation electrophoresis, respectively, suggesting that the patient may have had malignant B cells or plasma cell disease. Approximately 85% of plasma cell tumors can express heavy and light chains simultaneously, and approximately 15% of cases only express a light chain. Most cases do not demonstrate the expression of the whole B-cell antigens CD19 and CD20, and CD38 and Ig-related antigen CD79a and CD10 are occasionally expressed. Normal plasma cells comprise CD19+ and CD56/CD58– cells; however, malignant plasma cells include CD19– and usually CD56/CD58+ cells; therefore, the patient is likely to have had pulmonary primary lymphoma (PPL). The clinical diagnostic criteria for PPL are as follows: (1) clear histopathological diagnosis; (2) unilateral or bilateral lung, lobe, or main bronchial involvement, with or without hilar or mediastinal lymph node invasion; and (3) no lung or extrabronchial tissue or organ lymphoma within 3 months after diagnosis. Pathology biopsy combined with immunohistochemistry confirmed pulmonary MALT lymphoma. The patient had a symptom history of 2 years, bilateral lung involvement, no hilar and mediastinal lymph node invasion, and no other organ involvement at 3 months of follow-up, which allowed us to confirm our diagnosis of PPL. Negative CD5, CD23, and cyclinD1 also help differentiate between small lymphocytic lymphoma/chronic lymphocytic leukemia (SLL/CLL) and mantle cell lymphoma, while CD10 helps distinguish follicular lymphoma; therefore, patients are likely to be considered to have pulmonary MALT-MZL. Simultaneously, interstitial changes were seen on the patient’s chest CT scan, related rheumatic immunological abnormalities were improved, lip gland biopsy showed positive results, and primary SS (PSS) was considered.

The complete clinical data of this case and 47 cases reported in the China National Knowledge Infrastructure database are presented in Table 1. Women and men comprised 26 and 21 cases, respectively. Ages ranged between 25 and 79 years, with a median of 58.7 years. Of the 47 patients, 19 were symptomatic, and 15 had respiratory symptoms, including cough and sputum, with chest pain in 4 patients and dry mouth and eyes in 1 patient. Twenty-eight patients were asymptomatic, of whom 12 were detected by physical examination and one by routine chest radiography preoperatively for scalp-ulcerative basal cell carcinoma. CT findings revealed the following. One case had lesions on both sides, 9 cases had lesions in the left lung (upper lobe, n = 5; and lower lobe, n = 4), and 29 cases had lesions in the right lung (upper lobe, n = 15; middle lobe, n = 14; and lower lobe, n = 14). The diagnosis methods were as follows. Six cases underwent percutaneous needle biopsy, 11 cases underwent transfiber bronchoscopic lung biopsy, and 17 cases underwent open thoracic biopsy and video-assisted thoracoscopic surgery. The time from symptomatic or confirmed asymptomatic lung disease to histological diagnosis was 1 week to 4 years, with an average of 9.4 months. Moreover, 3 of 47 patients had a diagnosis time of >12 months due to nondiagnostic biopsy specimens, refusal to undergo biopsy or surgery, and the slow progression of lung lesions to the point that the possibility of malignant disease was not initially considered.

**Table 1:** List of case reports of pulmonary MALT Lymphoma.

Clinical characteristics of patients	Number of patients	Percentage (%)
Sex		
Male/female	21/26	44.7/55.3
Age (years)		
Mean	58.7	
Range	25–79	
Medical history		
Sjögren’s syndrome	2	4.3
Interstitial pneumonia	2	4.3
Clinical symptoms		
Yes/no	19/28	40.4/59.6
Respiratory symptoms	15	79
Chest pain	4	21.1
Dryness of mouth and eye	1	5.3
Initial diagnosis		
Pneumonia	10	21.3
Inflammatory diseases	5	10.6
Fungal infection	1	2.1
Pulmonary tuberculosis	2	4.3
Lung space occupying	12	25.5
Malignant tumor of lung	1	2.1
Primary lung cancer	4	8.5
Delay time		
Average	9.4 months	
Range	0.25–48 months	
Diagnosis		
TTNB	6	17.6
TTLB	11	32.4
VATS	17	47.2
Therapy		
Surgery	25	53.2
Combined chemotherapy(+/–R)	14	29.8
Surgery + chemotherapy	2	4.3
Chemotherapy + targeted therapy	1	2.1
Follow-up visit	5	10.6

MALT = mucosa-associated lymphoid tissue, TTLB = CT-guided percutaneous lung biopsy, TTNB = transthoracic needle biopsy, VATS = video-assisted thoracoscopy.

Approximately 5% of patients with PSS develop B-cell NHL, and > 50% of patients have MALT lymphoma.^[5]^ This implies that clinicians should pay more attention to patients suspected of such conditions in clinical practice, shorten the follow-up time, and conduct real-time monitoring.

Clinical predictors include permanent swelling of the salivary glands, splenomegaly, lymphadenopathies, and palpable purpura. The main biological predictors are the presence of cryoglobulinemia, lymphopenia (especially CD4 lymphopenia), low complement levels, and a monoclonal component in serum or urine.^[6,7]^ Simultaneously, the dose effect of disease activity can be detected because the risk of lymphoma increases with an increase in the EULAR SS disease activity index score.

Owing to the low incidence rate, the best treatment mode for pulmonary MALT lymphoma lacks the support of evidence-based medicine. Currently, the available treatment methods include surgical resection, chemotherapy, immunotherapy, radiotherapy, and observation; however, owing to the lack of site-specific guidelines, the best first-line treatment for pulmonary MALT lymphoma remains to be determined. Whether the observation and waiting policy is beneficial to patients, particularly at an early stage, remains controversial. To date, no relevant randomized controlled trials have been reported.^[8,9]^ Studies by Lee, Haroon,^[8,10]^ and others have shown that complete surgical resection may provide effective treatment results for patients with low stage pulmonary MALT. Some scholars believe that radiotherapy is the first choice for a limited period, including local recurrence.^[11]^ Some studies have shown that extragastric MALT lymphoma is a potential systemic disease unrelated to the initial stage.^[12]^ Woo^[9]^ et al conducted a retrospective analysis of 61 cases of pulmonary marginal zone B-cell lymphoma (P-MZL) and found no difference in the time to progression between the surgical and chemotherapy groups. They suggested that chemotherapy should be considered as the first choice of P-MZL treatment to protect lung function and reduce the risk of hand surgery. Chemotherapy includes single-drug therapy (including fludarabine, cyclophosphamide, azathioprine, steroids, and rituximab) and combination therapy (including the cyclophosphamide, doxorubicin, vincristine and prednisolone (COP) regimen, the cyclophosphamide, doxorubicin and vincristine (CHOP) regimen, and the lenalidomide-cyclophosphamide, doxorubicin, vincristine, and prednisolone (R-CHOP) regimen). Patients who relapse after at least 1 CD20-based treatment may be able to undergo several recently approved nonchemotherapy options, including B-cell receptor inhibitors such as ibutinib (specifically approved in MZL) and immunomodulators, including lenalidomide and rituximab (FDA-approved for MZL and follicular lymphoma). Phosphatidylinositol 3-kinase (PI3K) inhibitors exhibit excellent activity in iNHL, especially MZL,^[9]^ but the effects of these treatments have not yet been demonstrated in evidence-based medical research.

For pulmonary patients with MALT and PSS, Thieblemont et al^[13]^ proposed that the ESSDAI and IPI scores are valuable prognostic parameters, and patients with high ESSDAI and IPI scores have a worse prognosis. Compared to patients diagnosed with autoimmune diseases or without such a diagnosis, they will not have a poor prognosis in terms of the time to progression.^[4]^ No treatment for SS is related to the beneficial or harmful effects of lymphoma.^[6]^ Fully considering these prospective prognostic factors can help clinicians treat patients with SS-related MALT using more appropriate methods.

## Author contributions

**Conceptualization:** Fang Liu.

**Data curation:** Fang Liu.

**Formal analysis:** Fang Liu.

**Investigation:** Rongfeng Qu.

**Methodology:** Rongfeng Qu.

**Project administration:** Rongfeng Qu.

**Software:** Chunmei Hu.

**Supervision:** Chunmei Hu.

**Validation:** Limin Yang, Chunmei Hu.

**Visualization:** Limin Yang, Chunmei Hu.

**Writing – original draft:** Limin Yang.

**Writing – review and editing:** Limin Yang.
